# Analysis and Experiment of Wireless Optical Communications in Applications Dedicated to Mobile Devices with Applicability in the Field of Road and Pedestrian Safety

**DOI:** 10.3390/s22031023

**Published:** 2022-01-28

**Authors:** Eduard Zadobrischi

**Affiliations:** 1 Department of Computers, Electronics and Automation, Faculty of Electrical Engineering and Computer Science, “Stefan cel Mare” University, No. 13 Str. Universitatii, 720229 Suceava, Romania; eduard.zadobrischi@usm.ro; 2 Department of Computer Science, Technical University of Cluj-Napoca, Gh. Baritiu St. 26-28, 400027 Cluj-Napoca, Romania

**Keywords:** pedestrian safety, safety driving, infrastructure-to-pedestrian, mobile communication, vehicle-to-everything communication, visible light communication

## Abstract

Current developments and the need for high-performance devices that provide safe and reliable communications present a future perspective by using visible light as an alternative solution that can substantially improve road and pedestrian safety. The daily use of smartphones is imperative; thus one can build on this premise a system dedicated to the aforementioned problem. However, the problem of the visible light communication channel (VLC) is highly dynamic and becomes extremely unpredictable in terms of exposure to noise sources. Developing applications dedicated to direct communications with infrastructure and vehicles using portable devices is becoming a challenge and at the same time a necessary solution. The article proposes the shaping of an emission–reception architecture dedicated to adaptive fuse light communications using OCC (optical camera communication) but also standard VLC communications using ambient light sensors via an Android application. This approach aims to provide a first step in shaping information-sharing applications using VLC communications. As far as we know, this approach has not been implemented in external VLC systems. The performance of the architecture and the application was demonstrated by practical tests that confirmed the capacity of the technology even if we are in the first stage.

## 1. Introduction

**Figure 1:**
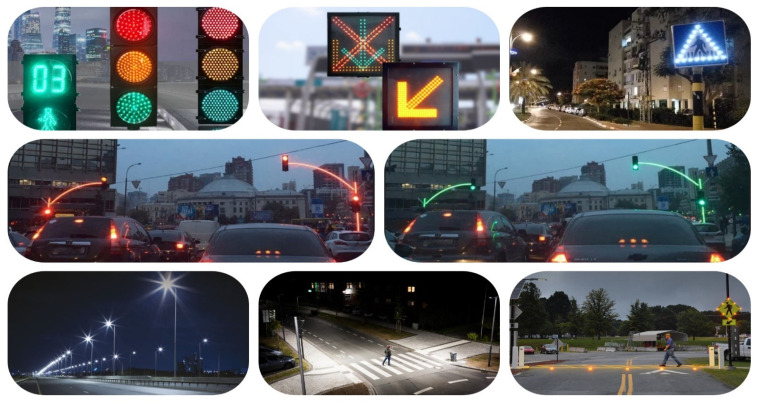
LED lighting sources installed in road and pedestrian infrastructures, capable of providing communications through visible light and optical camera communications.

Analyzing the current technological development and advancement, we observe an almost uniform growth in all fields, mainly in the technological one. Road safety policies and the continuous increase in the number of vehicles generate traffic congestion and at the same time create an environment conducive to road accidents involving pedestrians. This is a major cause of death according to the World Health Organization alone as between 2019 and 2020, over 450,000 people were extremely seriously injured in road accidents, and for many of them, it was not possible to intervene to save their lives [1,2]. According to the World Health Organization, a major cause of death worldwide among young people aged 14 to 36 is road accidents. These grim statistics are much more evident in general reports: more than 1.25 million people lose their lives each year in road accidents. According to the World Bank, between 20 and 50 million people are injured each year and suffer extremely serious injuries, some of whom remain immobilized for the rest of their lives. The aspect of mobility is fundamental for most activities undertaken by people, and the safety of transport systems and road safety are extremely stringent research topics in the automotive industry, car manufacturers, users and academic communities around the world [3,4]. We have reached a milestone of a century in the history of the automotive field and have taken part in the most important developments in the direction of driver control and analysis, the design of intelligent road infrastructure and other innovative services dedicated to the safety of pedestrians and traffic participants [5,6,7]. Therefore, this growing concern in the field has led to the implementation of new concepts that include state-of-the-art wireless communications that allow both vehicles and infrastructure to form transport networks, reduce the risk of accidents and improve the safety of pedestrians and other road users. The novelty elements that the new communication protocols based on LED light would bring take shape and can become normal in terms of lighting, being superior incandescent bulbs but also to fluorescent tubes in terms of efficiency, lifespan and even resistance to extremely adverse climatic factors [8,9]. LEDs are not only ideal solutions in people’s homes, but they are also used in street lighting systems, vehicle lighting systems and also in the case of lighting infrastructures for transport, traffic lights, and warnings (see Figure 1). Another extremely important advantage is related to the extremely fast switching capacity through which LEDs allow the use of the visible light spectrum in the context of information transmission. This feature can be implemented in vehicles, traffic lights, street lighting systems and pedestrian crossing warning messages, being an extremely versatile facility in addition to lighting capacity [10,11].

The involvement of visible light communication (VLC) technology requires a transmitter and a VLC receiver; they can facilitate the transfer of information and the data being modulated with the help of an optical carrier that has a VLC transmitter, and they are imperceptible to the human eye. The receiver transforms the modulated light into an electrical signal, and later, these data can be processed by different techniques [12,13]. In the case of using VLC for communications between infrastructure and mobile devices, we have two possibilities: processing images and using the front or main camera or the front sensor of light-dependent resistor type. VLC technology can have the ability to transform any light source of LEDs into a dynamic technology that facilitates timely communication with any device and through any device. Thus, VLC technology can facilitate direct communication by transforming any LED light source into a data transmission device [14,15]. The use of smartphones in the management of such a problem can substantially reduce the risk of road accidents and can combat the loss of human life caused by carelessness. Although the fifth generation (5G) has been introduced, there is still no stability in terms of data transfer rate, and it is not possible to take into account the reference speeds that have transfer rates above 10 Gbps [16,17]. Given the fact that there is a problem with the degree of massive connectivity of users through the Internet-of-Things (IoT) paradigm, we cannot have extremely viable expectations for RF technologies, and therefore we need an alternative. Existing RF resources do not have the necessary power and are not sufficient to support massive accessibility among consumers; in addition to this issue is the issue of bandwidth-limiting interfaces [18,19].

**Figure 2:**
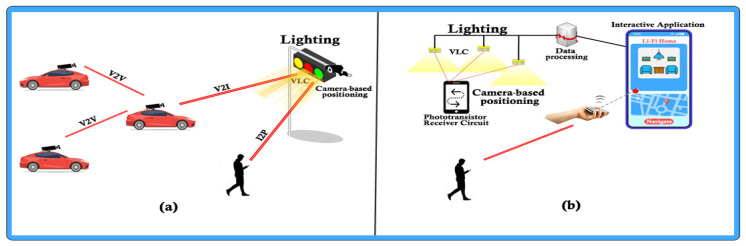
(**a**) The main scheme of V2I communications (vehicle-to-infrastructure) and (**b**) represent visible light communications based on optical camera communication and environmental sensors.

We must take into account that the optical spectrum has a problem, that of bandwidth, as it offers a wide range of frequencies (0.2–3000 THz) [20]. Therefore, wireless optical technologies (OWC), such as visible light communication (VLC), Li-Fi, optical camera communication (OCC) and top optical communication in an open environment, can make a significant contribution [21,22]. The existence of backhaul solutions that offer the possibility to optimize certain limitations, small cells and backhauling becomes a solution that offers a greater outsourcing capacity for 5G. Their usefulness is observed in implementation, flexibility and interference management but also delay management or overload. An advantage for OWC is that it supports much higher data transfer rates and communication distances that range from limited areas to extremely large areas depending on the spectrum available [23]. It should be noted that OCC does not support the densest link connections, which is caused by the hardware limitations of the cameras, which require the use of applications with low transfer rates, indoor simulations, low mobility and small scanning areas [24]. We must not neglect the fact that light-emitting diodes (LEDs), in addition to being used as emitters for OCC technology, can also be used for lighting (see Figure 2).

Both the cameras and the front area where the proximity sensors of light are found are embedded in smartphone devices. They are ideal for creating a wireless communication platform, saving installation costs, power consumption and hardware for receiving information. Such a solution could also facilitate the exchange of information between devices within the platform without any problems or interruptions of connectivity or limiting the number of users [25]. Perhaps the main problem is caused by the existence of several types of operating systems for smartphones (Android, Windows, iOS, BlackBerry OS). Thus, the same application that would facilitate direct communication between users could not be installed on all devices, requiring the development of customized mobile applications. In this case, the largest number of users of an operating system use Android, and thus they are part of the perspective of developing a solution. We believe that OCC is becoming a promising technology in both indoor and outdoor applications, facilitating people monitoring, intelligent lighting, artificial intelligence, augmented reality, image processing, detection and location [26,27,28,29].

The article comes with a solution dedicated to communication between infrastructure, vehicles and pedestrians by using visible light, OCC and light ambient sensors to prevent the loss of human life caused by carelessness. The article highlights the importance of mixed communications and the use of smartphones in managing extreme situations. The creation of an Android application to process the signal with the help of the camera and the photoreceptor with which most of the phones are equipped outlines another direction through which the research dedicated to road safety can be extended. Section 2 addresses the existing problems and solutions in the field of road and pedestrian safety with the help of mobile applications, highlighting the challenges and solutions presented by the literature. Section 3 presents the proposed system and the application developed in the mentioned direction, including hardware and software components and experimental tests. Section 4 provides a broad discussion outlining the results obtained and confirms the usefulness of this solution, as well as a clear direction for further developments, while Section 5 focuses on formulating relevant conclusions.

## 2. State of the Art in Optical Wireless Communications

### 2.1. The Contribution of Optical Camera Communications in the Processes of Information Transmission

**Figure 3:**
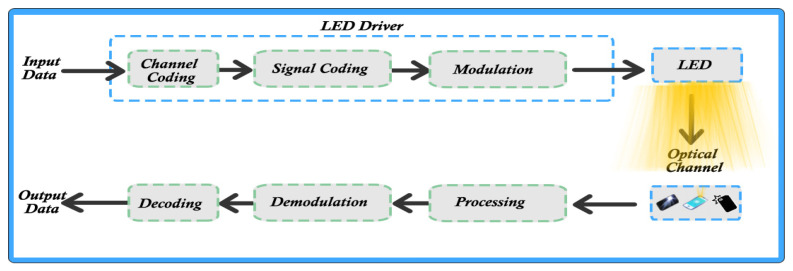
Modulation and demodulation scheme for VLC: mobile communications.

Current studies and developments in the presented direction have an extremely rapid succession, but a detailed and comprehensive study on OCC technology can be found in [30]. This section presents the most notable developments both in the direction of OCC systems and their use in the implementation of complex solutions. OCC can also include LEDs and image sensors that can be used for both transmission and reception. We emphasize again that almost any application uses LEDs for interior lighting, street lighting, signaling, road lights and car headlights [31]. Fast switching between On/Off states allows extremely viable and much more efficient transmission in such applications. Using this property, LED light can be modulated using high frequencies and several types of modulation. Figure 3 shows the block diagram of an OCC system, which uses both the camera and the front of the phone for communications [32,33].

Therefore, the coding process of the optical channel is performed to detect and correct the error element while the transmission process through that channel takes place, having noise and interference that are induced by ambient light sources for the interior. When we use the same process for the external environment, things are much more delicate because parasitic light and disturbing sources are present everywhere. The first solution used by most research groups to solve the stated problem was the Manchester coding which helps in the process of eliminating the flicker of light and reducing losses [34,35]. Although its use reduces certain aspects, sometimes the changes in the light of an LED are seen by the human eye when the luminescence exceeds the threshold of 200 Hz, a threshold considered safe [36]. Another aspect is that data are encoded and used to modulate the LED light at much higher frequencies than a camera could detect; thus there is the option of using the application that uses the front area where there is a photoreceptor [37,38,39].

The responses in the case of photoreceptor pulses are indirect measurements of high-intensity LEDs in order to generate flashes with a duration between 0.05 and 1.0 ms, these are mediated at 100–500 pulses, with a repetition rate of 1–2.5 Hz. When the peak wavelengths of the LEDs are located in the immediate vicinity of the UV threshold (360 nm, at half the width of 15 nm) or blue (470 nm, representing 22 nm) in the spectrum. Thus, we have a maximum spectral sensitivity of photoreceptors on the blue and green areas that tend to be 420 nm and 550 nm, at 480 nm relative sensitivity with about 0.30 and 0.5 [40,41]. This spectral sensitivity at the peak of the receptors sometimes reaches 348 nm, with a relative sensitivity of 360 nm. The aspect of source attenuation must be taken into account, as well as the attempt to find a way to effectively calibrate and capture quantum light for quality information processing when talking about individual photoreceptors, such as smartphones. It was observed during the studies that the noise in the photoreceptor area indicates individual quantum events, and considering the environment, there is the possibility to operate in a reliable and viable way after it is mediated with the environmental conditions. Adaptation in the case of low ambient light facilitates transmission, observing an increase in noise in the dark every few seconds, causing flashes at 10 ms (medium intensity), a factor that attenuates the amplitude limitation of the peak response to <10 mV. Therefore, we can deduce that the use of both the front area of the mobile phones and the camera is absolutely necessary to achieve compliant and stable communication [42,43].

Thus, the data encoded by the front of the smartphone use LED light at a much higher frequency than a camera can detect. At the moment, the image sensors of cameras demonstrate a limited capacity in detecting clear answers that have frequencies up to 6–7 kHz [44]. The tests so far have been based on real-time processing using smartphones, which is also quite difficult due to camera limitations and low processing power. However, the mixed use of cameras and photoreceptors in the front area of mobile phones can substantially adjust the way data are processed and transmitted. Even if the technological advance in this field is extremely difficult, this technology can have an enormous contribution to future IoT paradigms, especially in terms of security and high connectivity of users. Numerous experimental works [45,46] in the direction of OCC have been highlighted with their objectives and achievements in the direction of individual implementations. The usefulness of the solution in which a mobile application communicates directly with the infrastructure and vehicles can be declared viable only by the way it meets certain characteristics and provides satisfactory results. Most of the experiments performed by the research groups were limited to external scenarios and used single-lens digital reflex cameras (DSLRs) or portable devices for MATLAB processing and analysis [47].

Table 1 shows the OCC reference implementations with notable results, using smartphones, but also DSLR cameras, highlighting the problems or limitations they faced. The most relevant systems tested in the OCC direction based on OCC used combined modulation schemes and ON/OFF coding in the form of phase shift subsampling (UPSOOK), and the processing used multiplexing with a division into wavelengths (WDM) [48,49]. These aspects have reached quite decent distances near the communication area of 1.5–2 m, but the transfer rate is between 378 bps and 480 bps [50]. These limitations can also be found in the external environment, in some places being threshold problems and even saturation of the communication channel in the conditions of parasitic environments. The processing with the help of smartphones is a delicate problem due to the limitations that the hardware part of a phone has and what the software imposes.

### 2.2. The Contribution of Communications Based on Visible Light in the Transmission of Information

These visible light communications (VLCs) represent the use of the visible light spectrum (VL), between 380 nm and 780 nm, to obtain wireless data communications. Therefore, the data transfer part is performed as an additional functionality; in addition to the main purpose of lighting, both transmission and reception can be performed [56]. As for VLC, the data are modulated on the instantaneous power of light, in its simplest form known as on–off keying (OOK). In terms of data reception, they are extracted with the help of light detectable elements, photodetectors and cameras. In the case of the photodetector, it uses a reverse-bias photodiode, which is included in a transimpedance circuit, then provides an electrical signal directly proportional to the power of the incident light. In some cases, the photodetector element is replaced with systems that include cameras [57]. In this case, the extracted data are obtained by complex image processing techniques, and the performance of the system is directly proportional to the speed and quality of the camera.

Regarding the IEEE 802.15.7 standard dedicated to short-range wireless optical communications using the visible light spectrum, it was officially launched at the end of 2011. The current version of the standard can cover the physical layer (PHY) but also access control (MAC) [58]. In accordance with the standard, data transfer is performed by modulating the light intensity of optical devices. Therefore, the issues associated with high-resolution flashing and grading are becoming a priority direction for studies. Following the standard also takes into account mobility issues, damage to connections due to noise and interference with other light sources. Consistent with the developed applications and the required transfer rates, the IEEE 802.15.7 standard comes with approximately three types of PHY layers [59]. The first PHY is dedicated and designed for concepts of external applications with low transfer speeds and OOK use. The modulation was performed for the variable pulse position (VPPM), the data transfer rates being between 11.67 kb/s and 267 kb/s. PHY II and PHY III were designed and proposed for indoor applications with moderate data transfer speeds, with speeds between 1.25 Mb/s and 96 Mb/s [60].

PHY II uses OOK and VPPM; in the case of PHY III, it is used in CSK (color-shift keying) applications. The CSK process can be performed with the help of multi-colored LEDs and photodetectors of selective type depending on the color or with optical filters. Under the given conditions, the visible light spectrum is divided into seven bands, then the data are coded using various combinations with three colors, all done according to mapping rules. Those three physical layers coexist but cannot be used simultaneously [61]. Even though indoor applications are often the most versatile and more foldable for this communication protocol because the standard stipulates the use of the optical clock frequency with rates between 3.75 MHz and 120 MHz, when it comes to indoor applications externally, the optical clock rates are below 400 MHz [62,63]. Much lower frequencies must also be taken into account because LED light for street lighting or traffic lights requires much higher currents, and the switching time is much slower. Regarding OOK applications, this standard specifies the use of a 200 kHz optical clock, and in the case of VPPM, an optical clock frequency of 400 kHz are used. These clock rates have been taken into account to prevent interference with other light sources; the latter could generate harmonics that can have frequencies of several tens of kilohertz. VLC outdoor applications also automatically involve extremely long communication distances, which allows the appearance of the distorted signal and the loss of the communication path [64]. The multitude of types of LEDs also brings into question the appearance of the characteristics for each of them depending on the field in which they are applied. The different types of LEDs and their properties depending on the area and field of application are shown in Table 2.

This presentation covers only a brief discussion on the mode and exposure of LEDs, those converted to phosphorus (PC-LED) and LED multi-chip type (RGB LED). In the process of making VLC transmitters, RGB LEDs and PC LEDs are the most common and preferred because of the fact that they are also found in lighting fixtures having double utility for both transmission and lighting, capable of producing white light.

In addition to these aspects, open environment applications are disturbed by the interference caused by natural light or other artificial light sources. In mitigating the effect of adverse conditions, the standard offers the convolutional coding (CC) variant superior to the Reed–Solomon coding, ideal for indoor tests. There is a multitude of tests and measurements in which schemes with separate RS and DC blocks were tested, subsequently improving performance by about 1 dB [65]. In a preliminary conclusion, the realization of fully functional applications in the direction of visible light communication systems dedicated to the external environment becomes a challenge and an extremely pressing point for the academic environment. According to the clarifications and research, we can highlight the fact that there is a lot of research that has been limited in how to apply this technology, especially the integration of mobile devices in direct communications with infrastructure [66]. According to Table 3, we highlighted the most important research in the analyzed direction.

We can also see from Table 3 that all the efforts of the research teams were channeled over long transfer distances, speeds and stability. The external environment is a supreme test for most VLC systems and their stability because of increased dynamics and unpredictable conditions, starting from climatic factors to parasitic light. According to studies, we can use and outline an Android application that uses the entire front of mobile phones in the process of data management. This application needs some prioritization on the hardware components of the devices and the communication to be conducted according to the urgency of the messages.

Regarding the management of a road safety application, the aspects regarding the detection, transmission and reception of information can be carried out according to the color codes. These methods include ways of encoding and decoding, filtering and sampling signals. Notable and visible elements that come with the novelty are related to the usefulness of the application and the purpose of development, which is that a smartphone user who does not have distributed attention and travels a route without being informed about the traffic or pedestrian situation is informed regarding the obstacle or danger to which it is exposed.

## 3. Experimental Data and Results

### 3.1. Usefulness of Camera-Based Optical Communications in the Detection Process

In the case of image sensors, they cannot have high-performance features or extraordinary image quality, and their behavior varies depending on different smartphones. Even the sampling rate of a room can vary from extremely low to very high. In terms of OCC performance, data transfer rate or distance are features that often depend on how the measurements were made and the quality or parameters of the smartphone cameras. There are rooms with much better sampling rates that can offer much higher efficiency, but these types of rooms increase the costs compared to the standard ones. Therefore, the frame rate is the number of individual frames that comprise every second of a video. In the case of shutter speed, this is identified by the period time for each frame that is exposed to light. Shutter speed is always represented by fractions of a second. In the case of the frame rate per second, cameras that are rated at more than 200 fps are classified as HFR (high-frame-rate) cameras, and those with less than 50–60 fps are classified as LFR (low-frame-rate) cameras. Promises for HFR cameras have extremely high data rates, but most cameras on smartphones are LFRs with about 20–30 fps in video recording mode.

In an OCC system, there is a light source, an LED used to transmit data depending on the type of modulation used. Camera-type sensors are also used to receive data for individual frame processing. Thus, the frames that contain data from the LED light are captured and processed following the processing algorithm but also by supplementing with information from the front area of the smartphone, where photoresistors are found. To avoid interference and parasitic factors, each frame is exposed to light only for a short time, and the camera shutter performs the control, as is the case with the photoreceptor, which in many cases saturates and has a much slower response time. Addressing, in particular, the field of road and pedestrian safety, many accidents with loss of life occur between 15:00 and 24:00 when in low-light areas reflections of light are formed that disturb visibility. The first phase of the analysis and simulations was performed in a controlled environment; later, the tests were extended in a dynamic outdoor environment. It should be emphasized that the variation in frames, the duration of exposure and the focusing time according to frames were analyzed. When the frame rate and the analysis process are linear, the processing is conducted smoothly, with the specification that autofocus is turned off. Starting with Android 5 (which allowed the camera app to be interfaced and programmed via an API), newer versions do not support full exposure control. There is a possibility for some devices that the secondary camera can be adjusted via an API through manual procedures. To have full access to cameras, devices must provide threads capable of enabling application mobility via the camera’s secondary API.

In the process of testing and implementation of the application, we used a smartphone device equipped with an image sensor with a nominal resolution of 9280 × 6944 equivalent to 64 Mpx, with an optical format of 1/1.72″. The frame rate was e 21 fps; the Chroma type was Tetrapixel. In the case of pixel size, it had a size of 0.8 um and was of ISOCELL 2.0 type with a MIPI 4 Lane RAW interface, all benefiting from Super-PD autofocus. The operating system used was MIUI 12, a software version obtained by overlapping, which is based on Android 10, being one of the most stable versions available. The development of the Android application was conducted in the Android Studio Ultimate platform, for which the Java language was used to outline the functionalities in the first phase. The application will later benefit from an upgrade, including in terms of language, moving to Kotlin for much better versatility.

#### 3.1.1. Techniques for Extracting Information from an LED Using Optical Communications

The current cameras contain CMOS-type sensors; they include automatic running mechanisms. The technique dedicated to sequential reading becomes the main feature of the cameras that benefit from the shutter, more precisely when the frames are not processed at the same time. Hypostases are analyzed by a quick scan, both horizontally and vertically, however different from a camera with a global shutter, in which case the scene is analyzed completely at the same time. A camera that contains a global shutter facilitates the sensor to be exposed only once to the light, being able to support the closing/lowering state of an LED on a single frame. If a camera has a shutter, the pixels in each row are exposed simultaneously at time T.

Therefore, an image captured using the shutter allows multiple exposures. It has the quality of obtaining several states for LEDs in a single frame, through the prism of each row gradually exposed to light. If an LED flashes, opens and closes depending on the bitrate modulated in binary code, the image contains a multitude of black-and-white gradients. In these circumstances, the bandwidths depend on certain modulated frequencies; however, the number of bands also depends on the distance between the camera and the LED.

**Figure 4:**
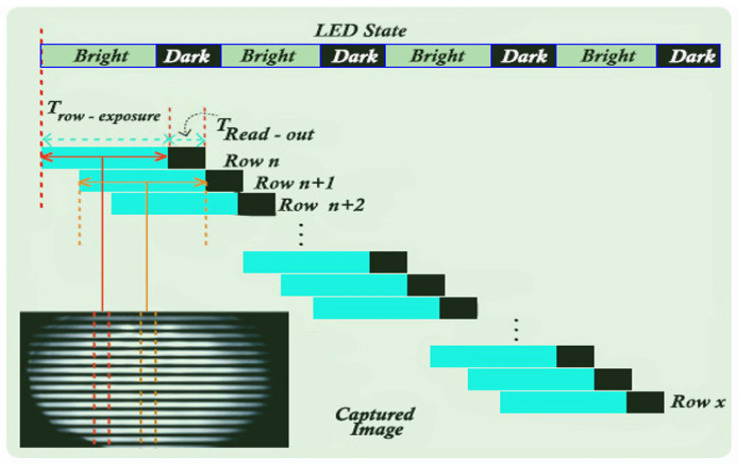
Diagram showing the technique of extracting information from an LED using optical communications based on the smartphone’s camera.

To be able to adjust and improve the ability to communicate over long distances, a photoreceptor is used. Figure 4 shows a model and an imaging technique of a room. In the example shown, the status of the LED is defined by blocks with black-and-white graphic layers, and the image captured from the LED is displayed only after going through a rotation process at 280°. It should be noted that the region is marked in the figure to understand the exposure time for each row denoted by *n* and for the upper rows denoted by *n* + 1. In another particular case, a transition region is obtained to the ON/OFF moment for the LED; this aspect iterates the state of change during the exposure.

#### 3.1.2. Real-Time Analysis and Processing through Optical Camera Communication Applications

**Figure 5:**
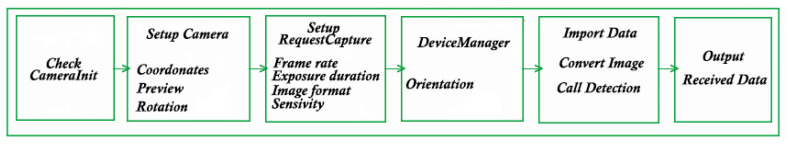
Dedicated OCC processing software architecture through the OpenCV library.

When it comes to real-time OCC applications, smartphone devices should have a fast way of processing information based on received images. The aspect in which the frame rate must be established initially and not include variations must not be ignored, and it is essential to conduct the processing during the whole duration of the frame. We assume that Rtf is the frame rate of the camera, then 1/Rtf is required to be the time limit for each frame made in real time. The literature states that for an OCC system that is based on a smartphone device with an Android operating system, an image processing application must be designed and developed to extract the data (see Figure 5). Whether we use OpenCV or another library dedicated to capturing images and extracting data, we need to outline an architecture. Therefore, a dedicated library with programmed functions that deal with the way of computerized extraction in real time can be outlined using Android Studio.

The most important aspect is the way and the permissions we build for the camera and other applications that are based on access to it. Therefore, the API must set priority levels for all elements that access the hardware component, from defining the size of windows to managing information. When setting the device orientation, we need to adjust the functions in DeviceManager. The image obtained from an LED can be processed to obtain information about the conversion and the required format. A key function is to identify the width of the LED strips. The need for an integrated library within the platform is intended to provide pixel reading functionality, being a threshold for object detection. The features of the new generation of Android systems describe the creation of execution threads that are extremely difficult to implement in the way requests are processed. Thus, it works to capture single frames and create buffer data sets. Creating a CameraManager class is required by priming multiple requests when they form a queue, which keeps the full frame rate constant. Data from images are encapsulated as objects and accessed using a class called ReaderImageCls using a TYUV_233_521 format initialized according to the operating system. Camera shutter speed management is controlled so one can focus directly on the area of the white LEDs and identify the region (RoI). The extraction of the bit stream that is transmitted is predetermined in the antenna, an aspect that is characterized as the main initial task.

#### 3.1.3. Projective Exposure of Regressive Transformations by Parameterizable Methods and Histograms

Therefore, when we need to detect objects and states, we use a computer vision application that provides the ability to observe the environment by performing data extraction and analysis. The specialized literature exposes the aspect that the detection of objects, states or pedestrians is an extremely challenging subject in terms of dynamic environments and the need for a series of parameterizable data on the conditions in which we need adaptable algorithms. The probability of a completely autonomous system that would behave ideally in the case of fluctuations is extremely low because, in the external environment, we have variations of images, light, scale, shaded areas, mixed dynamic background and uneven movement. Studies have shown that detecting objects or recognizing actions at night can be much more difficult than during the day. The most recommended method to perform qualitative detection is to use the concepts of key points which are extremely robust classes that describe specific properties for that image. It also performs changes in pixel intensity and orientation, T-junctions, corner abstraction or minimum size. The key points include a set of specific properties and analyze the intensity or orientation, reaching up to the use of 64 elements that contain unique descriptors. In this case, these unique descriptors are elements or key points from different images, which aim to indicate the same characteristic for the same area of the object.

In the analysis, we performed a binary process that represents the conversion of an image that has gray tones into an image with black-and-white characteristics using the thresholding method. The methods are different, but they have separately applicable characteristics and analyze the problem in the substrate. The threshold selection analyzed the described section and each gradient using the Otsu method [71], which outlines a histogram for images that it calculates according to an optimal threshold value. The Otsu method is based on a linear image pixel statistic consisting of two classes to binarize the image. This threshold is calculated using first-order moments, average *ave* and standard deviation *std*. An aspect is differentiated that an independent result of dimensions is obtained, and the histogram *H* is normalized as follows:

(1) $$H_{i}=\frac{n_{i}}{N},\;$$

where $n_{i}$ represents the total number of pixels of illumination, the level in the image is denoted by *i*, and *N* is defined by the total number of pixels in the image. In the case of the calculation of the mean and the standard deviation, they can be defined as follows:

(2) $$ave\left(k\right)=\sum_{i=1}^{k}i\times H_{i\;},$$

(3) $$std\left(k\right)=\overset{k}{\underset{i=1}{\sum }}H_{i\;}.\;$$

Thus, we define $s^{2}$ for each value assigned to *k* = 1, *n* to *n* + 1, 255, $s^{2}$, and we can calculate it this way:

(4) $$s^{2}\left(k\right)=std\left(k\right)\cdot \;\left(1-std\left(k\right)\right)\cdot {\left(ave\left(n+1*255\right)\cdot std\left(k\right)-ave\left(k\right)\right)}^{2}.$$

The value of *k* helps to maximize the function *s* from the optimal threshold value in the whole binary process dedicated to the image [72]. This is because maximization is achieved by separating the classes resulting from gradient extraction. We can determine the threshold *k* as follows:

(5) $$\;s^{2}\left(k\right)=\max\left(s^{2}\left(k\right)\right).\;$$

Gradual exposures must show a projective transformation in order to understand the analysis of the pixels in the image [73]. In the case of gradients and exposures, we established the image in a system of *x*, *y*, *z* coordinates on which the characteristics were matrixed.

**Figure 6:**
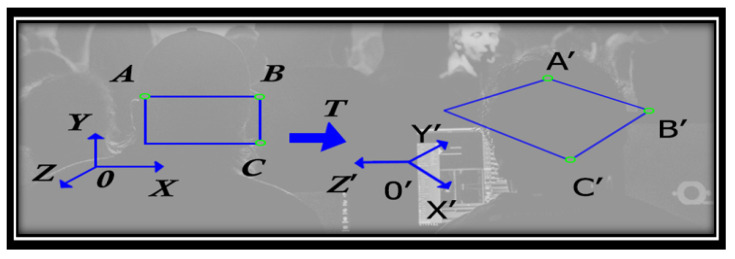
Exemplification of a projective transformation for distorting a rectangle—extraction capabilities.

In Figure 6, the representation of point *A* is part of the reference for $O_{XYZ}$ and *A*′ being the design characteristic in the $O_{X\prime Y\prime Z\prime }$, the plane for reference, where *A = TA*′. Then the points *A* and *A*′ are homogeneous, and their coordinates are ($A_{X}$, $A_{Y}$, $A_{Z}$) and ($A_{X^{\prime }}^{\prime }$, $A_{Y^{\prime }}^{\prime }$, $A_{Z^{\prime }}^{\prime }$), respectively. The matrix dedicated to the transformation is denoted by ***T*** *=* [$t_{ij}]$, representing a three-row and three-column irreversible matrix with a point coordination relation:

(6) $$\left[\begin{array}{c} A_{x}\\ A_{y}\\ A_{z} \end{array}\right]=\left[\begin{array}{ccc} t_{11} & t_{12} & t_{13}\\ t_{21} & t_{22} & t_{23}\\ t_{31} & t_{32} & t_{33} \end{array}\right]\;\times \;\left[\begin{array}{c} {A_{x}}^{\prime }\\ {A_{y}}^{\prime }\\ {A_{z}}^{\prime } \end{array}\right]$$

When the determination is made for all the elements contained in matrix **T**, the three points *A*, *B* and *C* and their projections are transformed according to the matrix exposure *A*′, *B*′ and *C*′.

**Figure 7:**
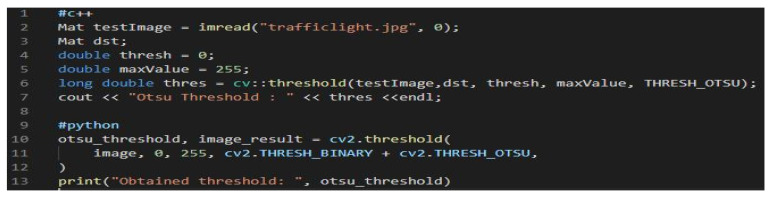
The code instance that imposes a threshold on internal class variables.

The images were converted by applying the Otsu method and reducing the gradient in the image by applying grayscale. This favors the operation of the thresholds imposed by Otsu in the case of two-dimensional images, thus converting to grayscale by syntax gray_img = rgb1gray (img) and plotting imshow (gray_img, cmap = “gray”), outlining that gradient map after exposing it as image transposed coordinates. The histogram generated by performing the exposed iterations offers the possibility of scattering colors in images, and the x-axis represents that scattering of colors. By the Otsu method, the pixels in the background are imperatively divided but also in the foreground with the successive imposition of a minimum color threshold to which a maximum color threshold is imposed. The imposed threshold must satisfy the variation within the inner class in relation to the threshold (see Figure 7).

**Figure 8:**
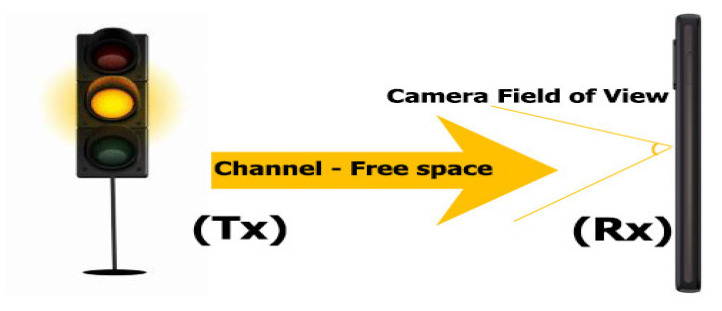
Illustration of an S-VLC communication system, which uses smartphones for reception and a street light for transmission.

**Figure 9:**
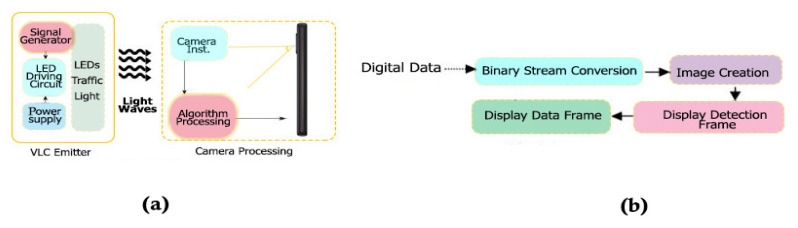
(**a**) Architecture of VLC emitter—camera processing. (**b**) Flow processing and binary stream conversion for frame detection.

Figure 8 illustrates a diagram of an S-VLC communication system, which uses smartphones for reception and a street light for transmission. The traffic light system transmits the information through the LED light, based on the wiring diagram shown in Figure 9a. The camera of the receiving phone captures the signal emitted from Tx. When the Tx light beam emits data, an encoded image creates a black-and-white gradient map demodulating the information. The camera at the Rx reception point captures the information in the visual field of view (FOV).

In the case of the block diagram for the Tx emission, this is described in Figure 9b. The input information goes through a binary conversion, then an image composed of several cells is used, and this cell contains an x number of pixels. The pixels in the same cell are assigned the same intensity. Subsequently, a stage of modulation of the communication signal is performed. At this stage, there are three ways to modulate the signal, and they are viable:

- Black and white cells/pixels: the outline of each cell, whether black or white, is a bit rate. When we have the modulation output in the form of an M × N matrix, it is allocated at intensity levels 0–255.
- Gradient cells/pixels (gray): for this operation, each binary model that is contoured follows a consecutive order with a quantity of 8 bits, being in turn represented by a decimal value 0–255. The output is also according to the matrix expression M × N.
- RGB cells/pixels: for this color format, most of the pixels are represented by the following three color components, at different intensities between 0 and 255. Therefore, for each cell, an input data stream is represented for those bits. The differentiation is made when the output has the shape M × N × 3, having a three-dimensional shape. The dimensions of the display matrices that are generated depend very much on the screen size of the device from which the measurements take place. If the amount of data is extremely large, then the data are divided into much smaller streams, and there is an image conversion.

Due to the inability to access the road infrastructure, the measurements regarding the ability to resonate with the proposed solution were evaluated according to the same characteristics presented outdoors, only in a controlled environment. In the case of VLC communication on the outside of its implementation in a traffic light system, it is not allowed, or the installation of light displays on the main arteries of some traffic signs is contrary to the legislation in force. That is why for the second stage of the tests we worked on creating miniature city intelligent intersections and lighting systems based on communications of this type.

Therefore, the distances for the interior scenarios were kept, and according to the presentations, the Euclidean distance was compared with the key matching points for a projective framework in the calculation of the transformations. Therefore, the next stage was the one in which the frame deformation and the data analysis were performed by quantization calculations and information binarization. According to the specified literature, we finally had the data set initially transmitted as a comparison frame T (x) by calculating the correlation between them. Thus, the following iterations refer to the deformation of the data frame, by comparing the initial frames and the quantitative binarization in determining the correlation.

**Figure 10:**
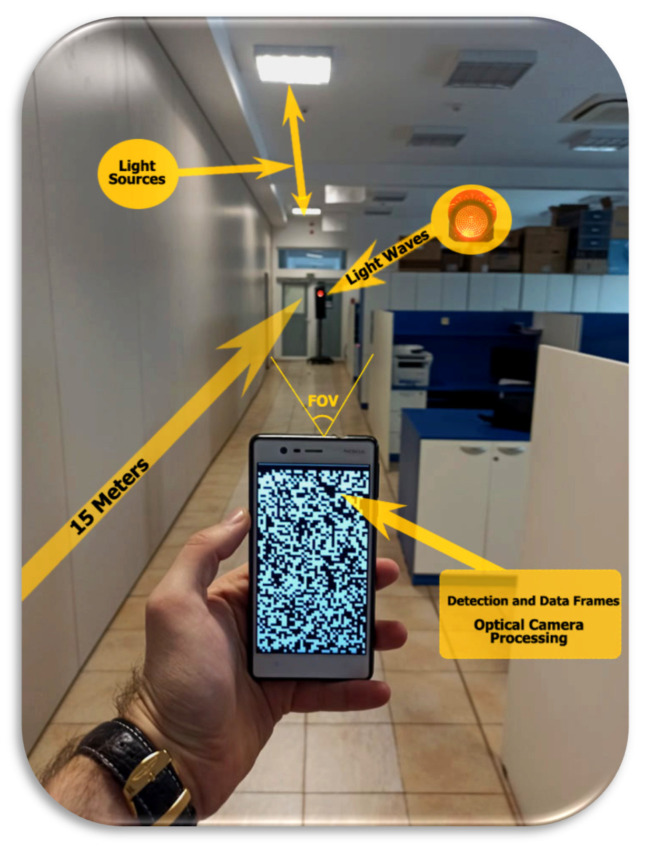
Detection process in an indoor scenario with disturbing lighting sources over a distance of 15 m.

In the descriptive Tx detection process, there is a possibility of losing the path and background noise to disturb the data quality. This similarity is found in most communication systems; loss of path and disturbance of ambient light or light sources can perforate communication and disrupt the connection. Therefore, the speeded-up robust features algorithm can adopt a necessary and extremely robust detection technique in the recursion of the Rx process. It is invariant about the detection scale and allows a wider range of applicability but also an extrapolation in terms of the visual field. It benefits from pixel-perfect accuracy and has a descriptor calculated for each key and point, an aspect that gives it uniqueness in the way it identifies data (see Figure 10).

The exposure of the key points found in the detection frame needs a pre-learning process; once those frames have been recorded, the matrix prediction of the transformation is calculated, later being used in the correction process. We need to keep in mind that a binary process and a symbol interface are needed to establish the analyzed pixels compared to the lux-ambient brightness level and the distance between Rx and Tx; cell capture and recording may be obstructed. Binarizing all the pixels in the image is a necessary solution to be able to obtain a granularity and a smoothing of the pixels to reduce the mirroring effect of the received signal. The information content is represented by black-and-white gradient images; in a standard initial process, they are captured in the form of RGB, and each pixel in the image has a representation of three types of component color intensity. The conversion is in gray tones and returns pixel by pixel until the degree of illumination level is positioned on a range between 0 and 255. In this conditioning, the initial process is constrained by restoring states and iterating states to reduce errors. In conditions where the distance is quite large, the irradiation of the VLC signal decreases substantially being influenced by the backlight having a low SNR. Thus, these cases lead to signal irradiation that can reach 0.076 µW/cm^2^; the reference is taken into account based on optical irradiation measured at distances of 1 m. At a later stage, communication is improved by introducing an external filter to reduce the field of view. For distances up to 15 m according to the literature, FOV is compatible with mobile applications because the angle between the traffic light and the receiver is less than 3.5°.

**Figure 11:**
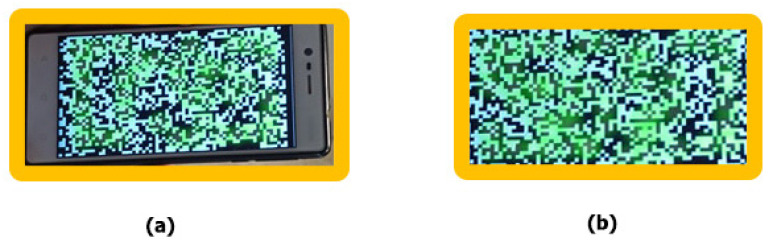
Determination of key points by detection frames; (**a**) reception area, (**b**) image in original format.

Binarization as a standard process depends on the threshold and the performance formed between the direct connection with the power of ambient light. Therefore, to be able to expose data as real as possible and as close as possible to something true, the Otsu method was used for threshold comparisons and passing the level of ambient light. If we assume that the total number of recognized pixels is found in the main mapping, then RX should scan the entire frame in a set order, from right to left and from top to bottom, to reconsolidate the reconstruction process. In the initial cells, we had a percentage of about 60% of the total number of pixels on the black gradient; the remaining pixels were converted later. The last step was to turn the entire image into black pixels and the final illustration of the messages through the quantization process (see Figure 11).

**Figure 12:**
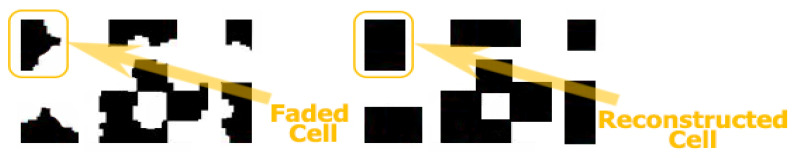
Before and after the process of constraining a data set (quantization).

As a result of the measurements performed, the variety of transmission and the proposed distance do not show disturbances, and in the conditions in which a fixed position is maintained for the mobile device, the movement on the communication line cannot be disturbed. A captured background image that does not contain relevant objects is contrasted by focusing directly on the communication path. Both Rx and Tx were gradually exposed to various types of lighting conditions, gradually using more panoramic lighting with fluorescent lamps and more precisely ambient font noise. The cameras of the phones were used to maintain the Tx image; the cameras used have an optical stabilizer (OIS) and automatic adjustment with a 64-megapixel image sensor (see Figure 12). The filtering stage retains only those quadrilaterals that contain the necessary characteristics; calibrating the position determines the respective court extremely precisely. Bit rates of approximately 11 kbps were obtained with an exposure of 2 at an interval of 0.7 ms, which can be adjusted by the method used or the encoding type. The experimental evaluation can be defined as follows: the performance was shown by the success rate of image transfer and processing (BER); in this case, an error rate of approximately 10^−3^ was obtained by correlating the data between transmission and reception.

### 3.2. Utility of Environmental Sensors in Mobile Telephony in the Direct Communication Process

Experiments performed in a controlled environment showed aspects of the ambient light sensor that detect light differently depending on the background of the area in which the device is located. In what follows, the aspects obtained in the measurements are highlighted, and it is underlined that depending on the intensity, the ambient noise can increase the bit error rate (BER). Therefore, in this case, an algorithm was optimized and created that would configure the adaptive sensor to adjust the minimum and maximum threshold values that cannot interfere with the data transmission. The initial sensor reads the ambient lights and then creates a matrix to observe certain characteristics. Subsequently, the application is channeled on the minimum and maximum values observed in a matrix. Therefore, the application identifies the minimum and maximum values that set a minimum luminance threshold, and then the application displays a warning message regarding the color, distance and other information received. To completely rule out interference, the situation must be managed by an LED configuration with a stable luminance in the broadcast areas.

The long-distance and angle calibration process includes aspects that can affect the demodulation process of the LED. When configuring the ambient noise, an LED can transmit binary data approximately 8 bits of 0, then it can resume a new cycle with 8 bits of 1. The application creates observational data from the nearby area to the distance when it can still detect the LED light. After the distance calibration process, the LED can transmit the same numbers of 0 and 1 in binary code to be able to calibrate the sensor at different angles, having that FOV in which the quality can be differentiated by the angles from which the light comes. The Android application asks the user to change the position of the device from 45° to 145° to take landmarks in future readings. With all these observational data, iterations can be run for the minimum and maximum luxury values in setting the threshold for the logical states of 0 and 1. Following the presentation of the application and the configuration we made for the indoor and outdoor environment, we used a 6.5-inch mobile device, on which the Android application developed was installed to calibrate the environmental sensor and setting threshold values as later this would determine if the illumination is in the range of 0 to 1. Subsequently, the application should show whether the read thresholds identify a light that is in the spectrum of those that are identified as dangerous (red, yellow, or strong flashes of the headlights can affect data analysis, and the display is limited only by their quantity).

**Figure 13:**
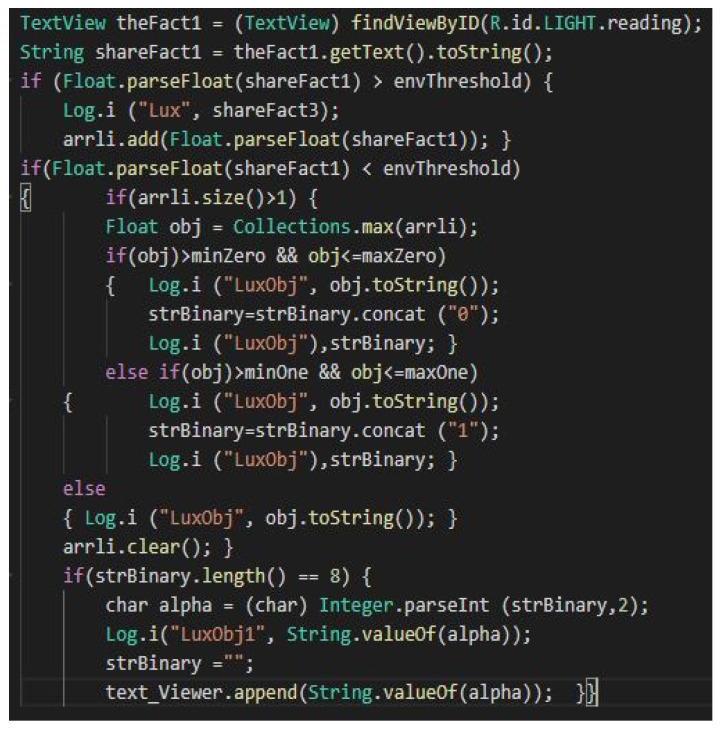
The structure of events for extracting binary characteristics from light intensity.

In order to obtain the data, we converted the objects into strings, based on the specific formats iterated in a string. According to the sequences in Figure 13, we observe the general use of string conversion methods formed to *Str*. When an object to be inserted after reading is not found in the procedure string, it is called to perform the conversion. If the concatenation index is 1, then the value is inserted into the string. The string is limited to 8 because larger messages can make communication, analysis or processing more difficult. The size is sufficient according to the nomenclature dedicated to road warning messages. After setting the length and characters of the message, we can move on to the next step when comparing events with the existing data set and parameters in the application.

**Figure 14:**
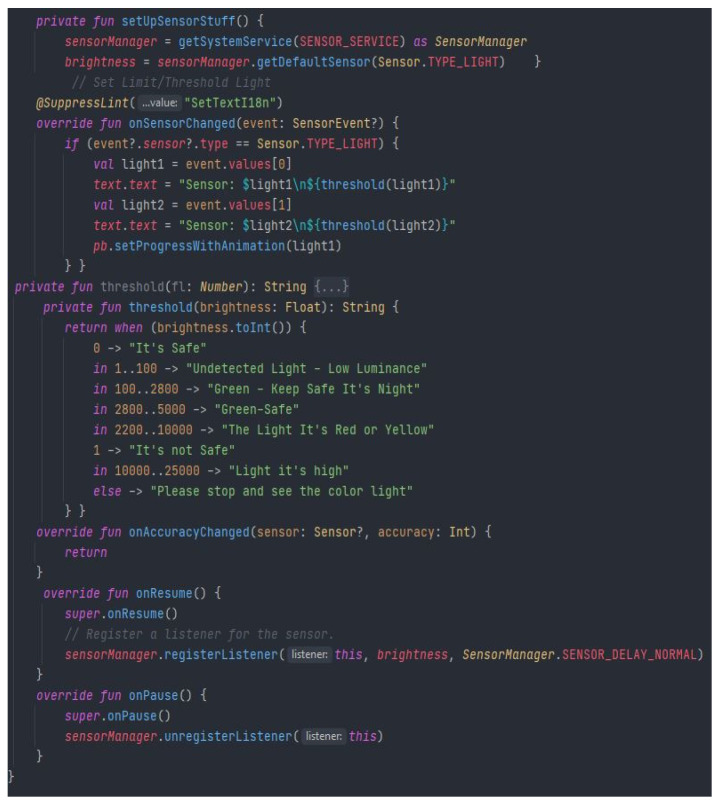
Structure of light intensity processing ambient sensor and setting safety thresholds.

Thus, after constructing the identification process and defining the light intensity, each data set is parsed, and an object defined as luminance with values of 0 and 1 is created following the concatenation of the binary string. The light intensity thresholds can be seen in Figure 14, which have predetermined values depending on the degree of danger that a certain intensity could constitute. These thresholds are set for the average sensor between 0 and 25,000 lx, compared to the headlight or daylight that falls directly on the photoreceptor. After prioritizing these features, the application modulates and decodes the messages, as long as they exist, so that it is not a pedestrian safety zone.

Measurements at a distance of up to 10 m in ideal conditions offer a viable perspective in the use of pedestrian and road safety applications using proximity sensors or ambient light. If even in a controlled environment a BER rate of 0% can be achieved, this aspect is no longer in the foreground at a traffic light intersection and on the main road, in which case the use of the OCC application applies. We cannot discuss these transfer speeds being extremely low compared to data transmission via ASCII maps as the purpose of the study was to detect instances dangerous for pedestrians based on the variation of external light near pedestrian crossings. There are times when changing the angle and position of the smartphone amplifies or obstructs communication directly, which is explained by the flat surface of the receiving area that does not have a gradual FOV and can result in angles of 30°–45°–90° in some cases of total efficiency.

In the measurements performed in the laboratory, different angles were experimented with, and it was observed that the flat surface of an LED is 100 mm and was exposed in different poses, obtaining for the angle of 90° a brightness level of approximately 800 lux for bit 0 and 1700 lux for bit 1. Depending on how the position of the smartphone changes from 30 to 35° or 40° with a constant distance from the LED, the incidence level of the brightness decreases drastically. Thus, for bit 0, a value of about 200 lux was returned, and for bit 1, the approximate value averaged around 500 lux. In addition, in the internal measurements, the aspects regarding the transfer rates in the case of light are highlighted, which are negligible by the technique presented. The ASCII modulation technique that can transmit characters in binary codes 0 and 1 is extremely vulnerable. Frequency-shift keying (FSK) modulation transmits digital data in different frequency configurations of carrier signals. This modulation is extremely useful in the transmission of urgent messages and in the case of radio broadcasting, where it is necessary to transmit data as quickly as possible. Using ArduinoDroid, convertible iterations were created for the serial data of the light signals, which change depending on the value received. The secondary algorithm modulates the hash mapping data that come from the brightness of the LEDs and parses each ASCII character. This algorithm uses a different method for each level of intensity in about 255 sections.

We must keep in mind that many of the measurements made by the research groups were obtained by configuring the LEDs with FSK modulation, and thus the receiver records the preset and initialized values of the ambient light sensor. Thus, each flash is detected and the light intensity values are automatically analyzed according to the configured luxury value range, displaying only the associated ASCII characters. We need to keep in mind that data transmission without delay sacrifices the attenuation factor and the transfer rate. If the change in brightness frequency is not possible then the ambient light sensor will not be able to detect the intensity nor will it have the desired efficiency in terms of the effect of dimming and scattering the light. The cause of this anomaly is a much higher bit error rate (BER) and low efficiency, putting delay thresholds in LED lighting of about 50 ms; if we add this fluctuating delay every 10–15 ASCII characters, we obtain an extremely useless 200 ms time factor. During the external measurements, the reception was configured on the threshold of light intensities from 1 to 255 to be able to have data even from small intervals. A serious problem is that of the refresh rate and the saturation of the sensor; when the light is too strong, it fails to differentiate the ASCII characters, the demodulation being performed with a delay of about 5 ms. In the external environment, the following aspects were highlighted at distances of 5 m, 10 m and 15 m in a straight line from a traffic light area:

– Divide the brightness range between minimum and maximum equally for each character; the ambient light sensor will not be effective if it needs to analyze all 255 characters from a greater distance.
– TrafficLightI2P color and hazardous area identification returned tangible values regardless of background noise or brightness value.
– The measurements were performed at a time of day when the entire lighting system was at full capacity and created a disruptive factor in most of the areas analyzed.
– Messages sent from another device in the nearby area complied with the algorithm, and the transmission interval for ASCII and decoding characters did not exceed 10 ms.

**Figure 15:**
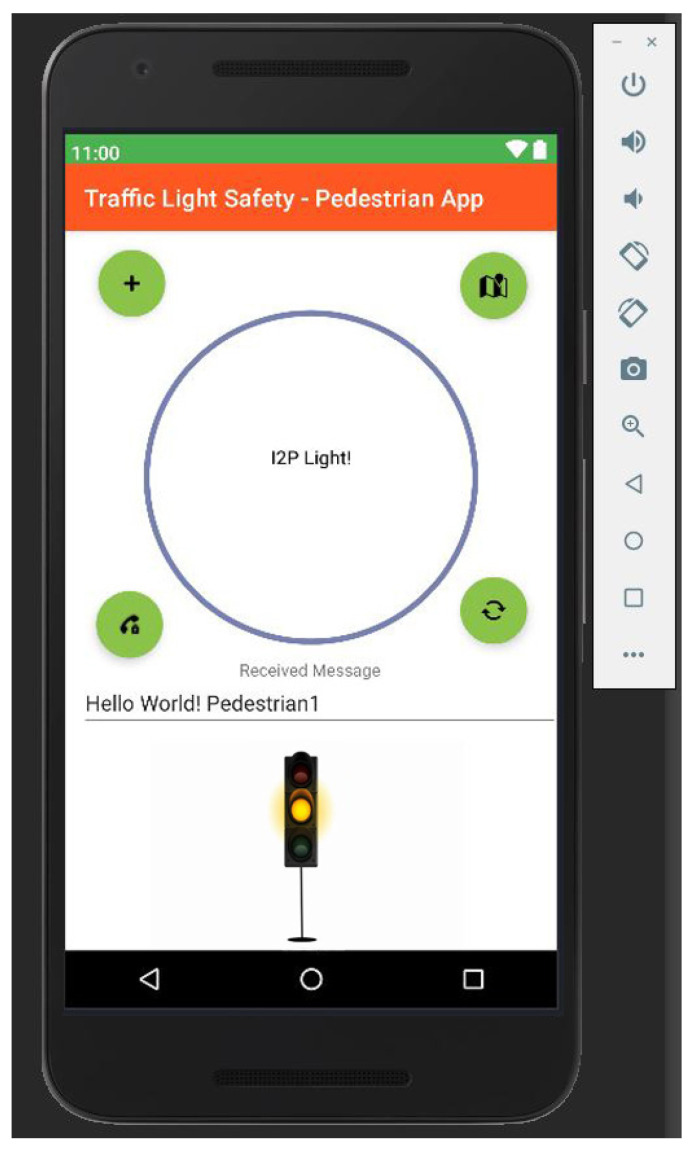
Android Studio interface dedicated to the color analysis of traffic lights and direct communication with other users. Pedestrian and road safety and prevention events.

As we can see in Figure 15, the application is structured extremely minimalist, with a relative layout, with a progress bar that collects information from sensors and classifies them according to the constraints created in the activity log. Regarding the identification of the traffic light colors, it was chosen to add a contrasting traffic light that changes its color according to the identified detection, with the mention that besides this aspect, the application also leaves alert-type messages. Messages are designed to warn the user and make them check the information. The center of the screen shows the data collected from the sensors and the warning messages; if things degenerate, there are predefined functions that automatically call the emergency numbers if the user does not close the information note within 10 s of its triggering.

**Figure 16:**
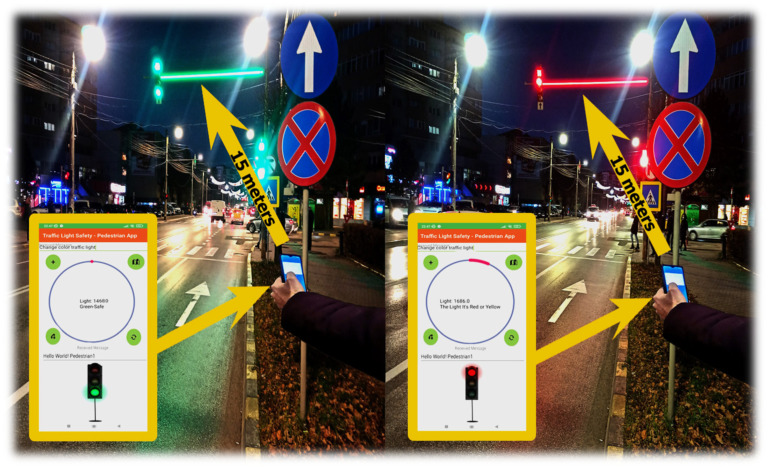
Exposure of the application in a dynamic environment, making measurements over a maximum distance of 15 m.

According to Figure 16, we exemplify the process of analysis and identification of light signals emitted by the traffic light system. The illustration shows the maximum distance at which the light intensity was identified, namely 15 m, enough to create a decision-making factor to prevent a major situation in which a pedestrian is involved without endangering their life. Therefore, analyzing the distance, we can show the aspect that the application and the researched direction offer perspectives in future developments with the mention that at this moment this type of communication can be viable only at night. The transmitted messages are received in the form of ASCII characters, displaying a warning message; considering BER, this is uncertain due to the mapping of the data for each bit exposed to light resonance. If we change the position of the device, we can have an area of incidence of +/− 10 mm, the BER being extremely high and the data inconclusive. According to Table 4, the information shows the light intensity that the device receives and the distance that it is at.

Depending on the amount of light received, we can show another aspect: in conditions of heavy traffic or taillights, we have a high degree of saturation and stray light. Being in an early stage of development, the goal was to demonstrate the ability to communicate through visible light using mobile devices. The thresholds set in the application have different iterative repetitive loops for each color gradient, thus obtaining a response from the extraction performed. Through the received light intensity, we can see that we also have a sufficient amount of data to be decrypted. There is also the possibility that the data may become false positive in terms of external lights, headlights, braking lights and street lighting. In this case, the use of optical filters is imperative when combined with hardware filters (optimized high-pass filter or low-pass filter).

The existence of a high degree of saturation or parasitic light can distort or congest the communication channel, but in this case, the application of an optical filter to eliminate the noise of the signal becomes necessary. This approach was also applied in the case of external scenarios between vehicles (V2V), having an 80 nm bandpass optical filter with a central wave of 645 nm. It was suitable for the given situation because it was also tested in conditions of exposure to light sources, including the red one redirected from the external environment (traffic lights, brake lights, street advertisements). This filter can eliminate up to 85% of ambient optical noise.

At the next stage, the inclusion of an optical decoder based on the PDA100A PIN photodiode is considered, but this version would mean the standard modification and the transition to an implementation that includes the outsourcing of the portable device and the inclusion of new modules. Thus, the optical decoder generates an electric current that depends directly on the power of the incident light, then the internal circuit transforms the electric current into an operable voltage so that information can be decoded. In this case, the analyzed information was shaped around the established intensities, only by using DroidStudio, and data-frame-type synchronization headers were created to be able to extract the data through the modulation used.

Therefore, the data presented provide a qualitative and quantitative perspective on the long-term implementation of this solution. Direct communication between portable devices and infrastructure is limited because most pre-installed components are in an adapted version and do not have the characteristics of commercial ones dedicated to industrial implementations. Currently, the results are satisfactory in the conditions in which there was no direct access to a traffic light system, this being the next step in which an autonomous intersection will be implemented that can communicate directly with traffic participants and pedestrians.

## 4. Discussion

There is a viable premise in both approaching VLC communications for mobile telephony with applicability in the field of road safety and the use of OCC for detection and analysis, and also data communication with applicability in the same field mentioned above. Most people own smart devices or smart mobile phones, and it is true that many of them do not benefit from superior performance and features in order to categorize this direction as extremely easy to implement. The main purpose was to demonstrate and show that by using the camera, we can have communications and data recovery can have a percentage of about 99% over a distance of 1–5 m. Depending on the increase in transmission distances, the bit success rate decreases and sometimes exceeds 50–60% over distances of more than 10–15 m, with the mention that the camera sensor has a resolution of approximately 64 megapixels, produced by Samsung. In the first stage, the results are satisfactory, but they are closely related to the performance of the cameras. The success rate of bits over short distances is remarkable, reaching 100% on a measured length of 80–100 cm. We cannot say that the binary process is a complete one and that it completely excludes the backlight or the illumination of the screen; solutions in these cases are the automatic adjustment and the calculation of the threshold for the mediation between the optimal illumination and the incidence area. The first stage was materialized by the Otsu method, and it was demonstrated that the performance of such a solution could be optimized even if there are disturbing areas or factors that negatively influence it. Direct exposure to ambient light and fluctuating threshold level +600 lux can disrupt the decision. We can also point out that there were trends in which the color black offered a strong shine compared to the white one, making a much more difficult transition in the process of schematic comparison. The geometric loading and setting of FOV in the process variation of the Tx rotation—where the devices were positioned in the same direction—were also performed. Regarding the secondary stage in which Android Studio and OpenCV conditioned with the use of ambient light sensors were used, the results are to some extent viable with the specification that in strong reflections there is a degree of saturation of the sensor. The purpose of this direction was clearly mentioned in the text of the manuscript. Due to the multitude of road events in which people lose their lives due to inattention and excessive use of mobile phones, outlining an application that can communicate information through visible light and interpret light frequencies as dangerous is imperative.

Therefore, the results obtained can be described as useful in the conditions in which a mobile device has an ambient light sensor without too much capacity at a low refresh rate and cannot even adapt directly to light. It cannot be compared to an experiment in which there is a VLC reception system based on PIN photodiodes with another FOV and completely different characteristics. Under the given conditions, decoding of the signals and identification of the light frequencies at distances of approximately 15 m from a traffic light system in a time of approximately 8–11 ms were obtained. Human reactions are usually extremely delayed, and the analysis process compared to a pedestrian’s decision change, in this case, reflects reality. Thus, in addition to identification, the application requires access to the phone’s vibration module and sound control to create a high-risk area alert activity.

## 5. Conclusions

The paper addressed the subject of optical communications by using cameras on mobile devices but also trying to communicate with existing sensors on these devices, namely the ambient light sensor. The investigations were carried out in a direction related to the fields in which VLC is applied; it is extremely important to be able to work with existing means, detecting images and decoding them and analyzing and disseminating information through visible light using decoding and signal processing methods. Projective transformations and quantization methods were used in addition to image processing and objective detection. The tests were performed under similar conditions in both directions, with the exception that only the results of the real checks were presented to support the cause. The results obtained underline the reasonable performance in which there could be standard road safety communication smartphones and infrastructure with the desire to be able to develop their capacity in the direction of communications between vehicles, infrastructure and pedestrians. The research is in a preliminary stage, and the existence of more ample results is sustained, but there is not a sufficiently solid premise at this moment to show within the same work. The proposed development is useful for short distances as long as the user is using a state-of-the-art smartphone with high hardware performance. The performance of these tests also depends on features such as refresh rate, saturation, resolution or focus. In the future, the studies and experiments will be extended in the direction of mobile platforms and the realization of simulations only in the external environment and during the day outdoors in conditions of extreme mobility.

## Figures and Tables

**Table 1:** Studies and implementations regarding OCC communications, limitations, challenges and results obtained.

Article	Direction	Achievement	Limitation/Challenges
[51]	Optical camera based on RGB LEDs using UPSOOK and WDM communication.	Transfer rates of approximately 140–150 bps and communication distances of over 50–60 m.	Simulations performed offline using the procedure in MATLAB. Use of a single LED RGB and much smaller capacity.
[52]	Using communication through the smartphone’s optical camera by modulating the intensity using four levels.	Transfer rates of about 10 kbps and a communication distance of about 2 m.	It cannot provide real-time processing. Using a single LED and limiting communication distances.
[53]	Using OCC and for coding, they performed the modulation by changing the color.	Transfer rates of about 8.64 kbps and a communication distance of about 4 cm.	Limited communication distances. Inability to support multiple connections through LED communications.
[54]	Improved optical camera communication systems using a freeform lens.	Using a freeform lens, the packet reception rate increased by 35% and the BER is reduced by 72% to a frequency of 5 kHz.	The distance for viable communication is much too short (160 lux at a distance of 1 m).
[55]	Indoor VLC communications using the smartphone’s camera.	The transfer rates were not noticeable being of the order of kbps and at distances of a few cm.	Mobility does not exist, and there is no support in this directive. Reduced communication distance.

**Table 2:** The characteristics of LEDs depending on their type, performance, complexity, band and cost.

	Complexity	Performance	Bandwidth	Cost	Practicability
RGB LED	Moderate	65 lm/W	15–20 MHz	High	Lighting
PC-LED	Low	140 lm/W	5–3 MHz	Low	Lighting
OLED	High	50 lm/W	≤1 MHz	Lowest	Display
μ-LED	Highest	-	≥300 MHz	High	Biosensors

**Table 3:** Results obtained in the integration of mobile telephony in visible light communications.

Article	Direction	Method	Results
[67]	PC-to-PC connection and transfer text, images using visible light communications.	Transfer information (the encrypted data through visible light, and receiver identifies and descripts the information).	The study was successful in sending the text and image text with accuracy of 100% and image 99% at a rate of 9600 kbps.
[68]	Vehicle-to-vehicle distance estimation using low-resolution camera based on visible light communications.	The work developed a high-speed and long-distance communication using VLC system and blue light LD.	The real-time transmission was 1.445 Gbit/s optical OFDM signal in 4.8 m underwater channel. The error vector magnitude was approximately 10%.
[69]	Transfer text and image, reception text and image using light from LEDs and a light sensor.	This work transferred the image and text to bit with Raspberry Pi platform and Python.	They successfully transferred one line of text with image 80% received.
[70]	Study that was based on communication using GaN micro-LEDs with E-O bandwidth (1.3 GHz for multi-gigabit visible light communications).	Based on the high-speed micro-LED, they demonstrated that a transfer rate of 2 Gbps and a BET of 1.2 × 10^−3^ can be obtained over a real distance of 3 m.	The results include a 4 Gbps system with multiplexing and orthogonal division in frequency and phase shift in quadrature and with a BER of 3.2 × 10^−3^.

**Table 4:** Results regarding the analysis, detection of colors, established thresholds, values measured according to distance and confirmation of message reception.

Distance (m)	Intensity Traffic Light—Red Color (lx)	Intensity Traffic Light—Green Color (lx)
1	4458.0	4812.0
5	3184.0	2445.0
10	2134.0	1957.0
15	1686.0	1468.0
